# Targeting individual cells by barcode in pooled sequence libraries

**DOI:** 10.1093/nar/gky856

**Published:** 2018-09-26

**Authors:** Navpreet Ranu, Alexandra-Chloé Villani, Nir Hacohen, Paul C Blainey

**Affiliations:** 1 Department of Biological Engineering, Massachusetts Institute of Technology, MA, USA; 2 Broad Institute of MIT and Harvard, Cambridge, MA, USA; 3 Center for Immunology and Inflammatory Diseases, Massachusetts General Hospital, Charlestown, MA, USA; 4 Department of Medicine, Harvard Medical School, Boston, MA, USA

## Abstract

Transcriptional profiling of thousands of single cells in parallel by RNA-seq is now routine. However, due to reliance on pooled library preparation, targeting analysis to particular cells of interest is difficult. Here, we present a multiplexed PCR method for targeted sequencing of select cells from pooled single-cell sequence libraries. We demonstrated this molecular enrichment method on multiple cell types within pooled single-cell RNA-seq libraries produced from primary human blood cells. We show how molecular enrichment can be combined with FACS to efficiently target ultra-rare cell types, such as the recently identified AXL^+^SIGLEC6^+^ dendritic cell (AS DC) subset, in order to reduce the required sequencing effort to profile single cells by 100-fold. Our results demonstrate that DNA barcodes identifying cells within pooled sequencing libraries can be used as targets to enrich for specific molecules of interest, for example reads from a set of target cells.

## INTRODUCTION

Intensive interest exists in applying single-cell genomic analyses including gene expression, chromatin accessibility, and DNA copy number variation to resolve differences between cells in a population. Pooled analysis of thousands of single cells is now routinely practiced by introducing cell-specific DNA barcodes early in cell processing protocols to produce a pooled library that is sequenced as a single sample and deconvoluted *in silico*. While such pooled experimental workflows are now a mainstream approach in life science research including cell atlasing efforts (1–8), these workflows do not currently enable cell targeting, even in cases when only a few rare cells are of interest (9–11).

As cell type and cell state discovery moves towards rare target populations (12–14), the challenge of identifying and accessing rare cells in pooled sequence libraries becomes increasingly important. In instances where rare cells are of interest, investigators must cope with applying extremely high sequencing effort or the sample loss and perturbation associated with enrichment by fluorescence-activated cell sorting (FACS), which ultimately limits the types of samples that can be processed (15). Here, we introduce a PCR-based approach to enrich pooled single-cell sequence library for reads from individual cells of interest. This approach enables investigators to selectively access relevant information out of such libraries with reduced sequencing effort. For example, cells that initially lack sequence coverage can be targeted for deeper follow-up sequencing and rare cell populations too small in quantity or too sensitive to perturbation for pre-selection by FACS can be enriched from the original pooled sequence library. Alternatively, the PCR enrichment approach can be combined with complementary enrichment approaches like FACS to target ultra-rare cell types.

Here, we apply PCR enrichment to populations of primary human B-cells, monocytes and dendritic cells from blood, which represent 15–35%, 10–15% and 1–2% of total peripheral blood mononuclear cells (PBMCs), respectively. We pre-enriched these populations by FACS using the following cell surface markers: B cells, CD19^+^ subset, from here on referred to as CD19^+^ cells; monocytes and dendritic cells, Lineage^–^(Lin^–^) HLA-DR^+^ cell subset, from here on referred to as HLA-DR^+^ cells. We demonstrate below how FACS pre-enrichment can be combined with PCR enrichment from large pooled sequence libraries to focus sequencing effort on an ultra-rare cell type of interest such as the AS DCs within the HLA-DR^+^ subset, which represents only 1–3% of human blood DCs and 0.01–0.06% of total PBMCs.

## MATERIALS AND METHODS

### Sample sourcing and FACS

This study was performed in accordance with protocols approved by the institutional review board at Partners (Brigham and Women's Hospital) and the Broad Institute. Healthy donors were recruited from the Boston-based PhenoGenetic project, a resource of healthy subjects that are re-contactable by genotype (16). The donors had no family history of cancer, allergies, inflammatory disease, autoimmune disease, chronic metabolic disorders, or infectious disorders. Each donor provided written informed consent for the genetic research studies and molecular testing.

For profiling HLA-DR^+^ and the CD19^+^ cells, PBMCs were first isolated from fresh blood within 2 h of collection using Ficoll-Paque density gradient centrifugation as described previously (17). PBMC suspensions were immunostained with an antibody panel according to the manufacturer's protocol (Suppliers listed in Supplementary Table S3) designed to target live HLA-DR^+^ cells and deplete other blood lineages (CD235a, CD3, CD4, CD8, CD19, CD56) or to target live CD19^+^ cells and deplete other blood lineages (CD235a, CD3, CD4, CD8, HLA-DR, CD56) (Supplementary Table S3). Cells were sorted in a solution of 1× PBS and 0.04% of BSA and resuspended at a concentration of 1000 cells/μl.

### Single-cell library preparation and target cell enrichment

Single-cell RNA-seq library preparation was performed with the Chromium Single Cell 3′ method (10X Genomics) according to the manufacturer's protocol. Pooled single-cell RNA-seq libraries were diluted and combined in equal volume with KAPA 2× high fidelity hot start PCR master mix. The final DNA template and total primer concentrations were 0.1 nM and 0.1 uM, respectively. For multiplex (10 – 15-plex) barcode amplification, forward primers consisted of sequencing adapters (62 bp) and cell barcode specific sequence (16 base pairs) whereas reverse primers were complimentary to the fixed truseq adaptor sequence. Hemi-specific PCR was performed with an initial hot start at 95°C for 5 min, followed by 25 cycles of (95°C – 0.5 min, 68°C – 1 min, 72°C – 1 min), and ended with a final 4 min extension at 72°C. The reaction products were confirmed on an agarose gel. As few as 15 cycles of PCR and lower annealing temperatures were also tested and produced good results, although care should be taken when reducing cycle number to ensure that sufficient product quantity is obtained to enable purification and any desired quality control steps prior to sequencing. Each PCR was performed in triplicate to assess replicability. The PCR products were then purified by SPRI (Agentcourt, 1:1 sample:reagent ratio) and quantified with the Qubit fluorescence assay (Qubit dsDNA HS Assay Kit, ThermoFisher Scientific).

### Sequencing and primary data processing

Target-enriched single-cell RNA-seq libraries were loaded at 1.8 pM on a DNA sequencer (Illumina Miniseq) where read 1 (26 bp) sequenced bases in the cell barcode and UMI and read 2 (124 bp) sequenced bases in the transcript. Primary processing of the raw data was conducted using the CellRanger pipeline (10× Genomics). Secondary analyses were carried out using custom Python scripts. The custom scripts used for secondary analysis can be found at (https://github.com/nranu/SC_enrichment). Replicate sequence reads were aggregated by unique molecular identifier (UMI) with secondary analysis operating on UMI counts. Any UMI that received two or fewer reads was removed prior to secondary analysis.

### Correlation analysis and Bootstrapping

Gene expression profiles of a given cell were compared before and after enrichment by computing Pearson correlation coefficients. Correlation coefficients were calculated using the expression profiles of targeted single cells in the enriched libraries and the corresponding expression profiles within the original library. One thousand Bootstrap read samples were then generated from each dataset to enable comparing pre-enriched single-cell datasets against themselves. Bootstrap samples of both pre- and post-enrichment data matched the read depth present in the pre-enrichment library for each cell. To determine the highest expected correlation coefficient values given the statistical noise from read and UMI counting, correlations were computed among Bootstrap replicates from the pre-enrichment data derived from the same cells.

### Principal components analysis (PCA) and clustering

Feature selection was performed by excluding genes detected in fewer than three cells and removing genes that had low coefficients of variation with a nonparametric Loess regression using a window of 33%. This selection identified ∼1000 highly variable genes that were well-represented in the dataset. Next, the UMI counts per cell were normalized by the median of UMI counts across all cells and log_2_ transformed with a pseudocount of 1 and finally, Z-transformed. PCA was performed with the original deeply sequenced library as a training set with the enriched data subsequently projected onto the components defined in analysis of the original library.

### Targeting putative AXL^+^ SIGLEC6^+^ DC (AS DC) cells

To identify AS DC ‘purity scores’, we used a previously described signature scoring system (11). Briefly, we assigned a quantitative score to each cell based on the overall expression of a pre-defined signature gene set after correcting for ‘drop-out’ effects that commonly characterize single cell data (10). The reported AS DC population purity score was based on the top 10 most discriminative genes previously reported: AXL, PPP1R14A, SIGLEC6, CD22, DAB2, S100A10, FAM105A, MED12L, ALDH2 and LTK. This ‘purity score’ was used to identify the most likely AS DC candidate cells in the HLA-DR^+^ 10X library. Note that not all of the 10 classifier-genes were expressed across the putative AS DC candidates in the 10X library, which could be explained by different dropout rates characterizing the 10X library and Smart-Seq2 libraries, the latter having been used in the original AS DC discovery and characterization study (11).

## RESULTS

### Target cell enrichment by multiplexed hemi-specific PCR enables a 100-fold decrease in sequencing effort

**Figure 1.**
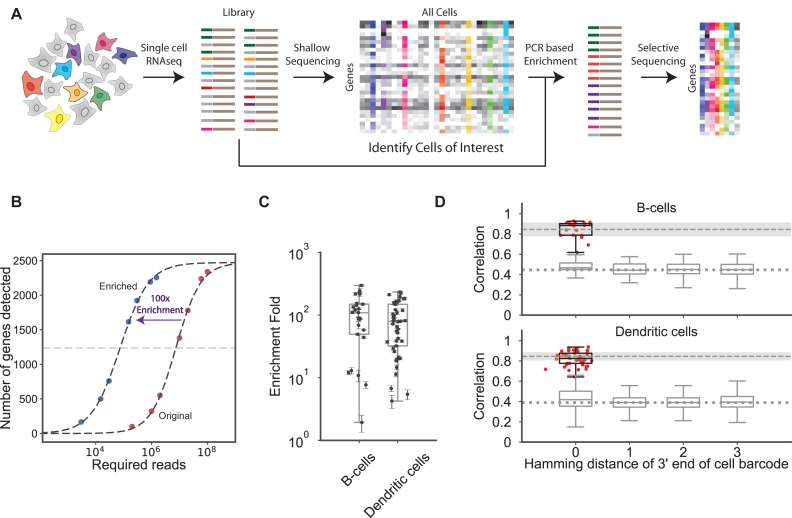
Targeted enrichment of single cells within a pooled RNA sequence library. (**A**) Workflow showing enrichment based on single cell barcodes on the 5′ end of sequence library molecules. Target cells (barcodes) of interest are identified based on shallow sequencing of the original pooled library. PCR with barcode-specific primers is used to create a new sequence library enriched for reads from the target cells. (**B**) Example enrichment plot for a single target cell from a multiplex-enrichment reaction. The original library was deeply sequenced as a control to identify gene expression profiles in the target cell. Enrichment fold is the fold-difference in overall sequencing effort to detect 50% of the maximum detectable number of genes. (**C**) Distribution of enrichment-fold values for 65 targeted cells amplified in multiplex PCR enrichments. (**D**) the pairwise correlation of gene expression profiles before and after PCR enrichment for CD19^+^ cells (top) and HLA-DR^+^ cells (bottom) libraries. The upper dashed line and shaded region in each plot represent the mean ± two standard deviations of Bootstrap replicates of the original gene expression profiles against themselves (which represents the best correlation achievable given the read sampling, UMI sampling, and distribution of expression levels across genes in these specific cells). Red points show the correlation for targeted cells (post-enrichment profiles versus pre-enrichment profiles for the same cell). Gray box plots show distribution of correlation coefficients for control (non-target) cells existing in the library (post-enrichment profiles of the subject control cell versus pre-enrichment profiles of all cells). The dotted line shows the mean correlation for the cell barcodes that had at least 6 mismatches at the 3′ end. Control comparisons are shown as a function of the number of mismatches (Hamming distance) between the six most 3′ base pairs of the 16-base pair subject control cell barcode and the six most 3′ base pairs of the 16-base pair barcode of compared targeted cells.

To preferentially amplify molecules representing target cells in the pooled sequence library, we carried out multiplexed hemi-specific PCR with forward PCR primers cognate to the barcodes of target cells (up to 15-plex tested; Figure 1A, Supplementary Tables S1,S2) and a common reverse P7 primer. To test the method, we targeted 19 cells in a sequence library representing 1760 CD19^+^ cells, and 46 cells within a sequence library representing 2397 HLA-DR^+^ cells. The forward PCR primers were designed to target the 16 base pair (bp) cell barcode appended to each cDNA 3′ tag sequence in the pooled RNA-seq libraries (Supplementary Figure S1). Target barcodes were selected to represent cells with higher (∼25 000) and lower (∼1000) counts of unique transcript molecules (Supplementary Figure S2, Supplementary Tables S1 and S2). We define target cell enrichment as the ratio in sequencing effort needed to access a specific level of information from a particular cell, here quantified as the number of detected genes. The libraries produced by our PCR protocol were enriched approximately 100-fold for the group of targeted cells (Figure 1B, C and Supplementary Figure S2). This enriched pooled library can be further sequenced to achieve deep coverage of high-quality target cells at far lower overall sequencing effort than would have been required in sequencing the original library. We found that the majority of reads in the enriched libraries corresponded to the targeted cells (Supplementary Figure S3, medians across replicates were 70–90%).

### Gene expression profiles of target cells are faithfully recapitulated after PCR enrichment

To evaluate the reliability of our method, we compared the expression profiles of cells targeted in the enriched libraries to each cell in the original library. RNA abundances in the enriched libraries quantitatively recapitulated RNA abundances from the original libraries, which were deeply sequenced and computationally resampled to provide matched control datasets for statistical comparison. We hypothesized that the base sequence at the 3′ end of the barcode PCR primer would be critical for maintaining specificity during amplification. Although 0.1% of cell barcodes share the same six base sequence at the 3′ end and are at risk for mis-priming events, we find that data from cells enriched in the CD19^+^ and HLA-DR^+^ libraries show expression profiles that are well-correlated with the corresponding pre-enrichment profiles (mean correlation of ∼0.82; as good as resampled replicates of the pre-enrichment profiles compared with themselves) (Figure 1D, Supplementary Figure S4). Further, the pairwise comparison of correlations across all targeted barcodes show the highest correlation for the intended target cell (Supplementary Figure S5). We observed a slight increase (statistically significant for the CD19 and HLA-DR subset) in the correlation to non-targeted cells when the 3′ end of the barcode has perfect complementary (hamming distance of 0). This effect is presumably caused by cross-priming, but does not significantly affect our final results as our filtering procedure (Materials and Methods) is designed to remove spurious UMI counts. In addition to barcode mis-priming, PCR chimeras have the potential to add noise to the measured gene expression profiles (BioRxiv: https://http://doi.org/10.1101/093237). We estimated that PCR-driven chimeras increase the UMI+gene collision rate by only a few percent above the statistically expected collision rate (Supplementary Figure S6). An additional source of noise can arise due to polymerase error during PCR amplification of UMI sequences, which might lead to inflated UMI counts. Although we did observe an increase in the number of UMIs at small Hamming distances (d_h_ = 1 - 2) that could be explained by polymerase errors, more than 99.9% of inter-UMI distance counts were at Hamming distances of 3 or more (Supplementary Figure S7), indicating that UMI inflation has only a minor potential effect on the data and that our filtering procedures likely exclude an effect. We note here that noise from all four sources: shared 3′ barcode sequence, statistical UMI+gene collisions, PCR-driven chimerism, and UMI sequence errors can likely be reduced by increasing the barcode/UMI complexity and redesigning the primers used for enrichment.

### Principal components analysis results in congruent cluster assignments

**Figure 2.**
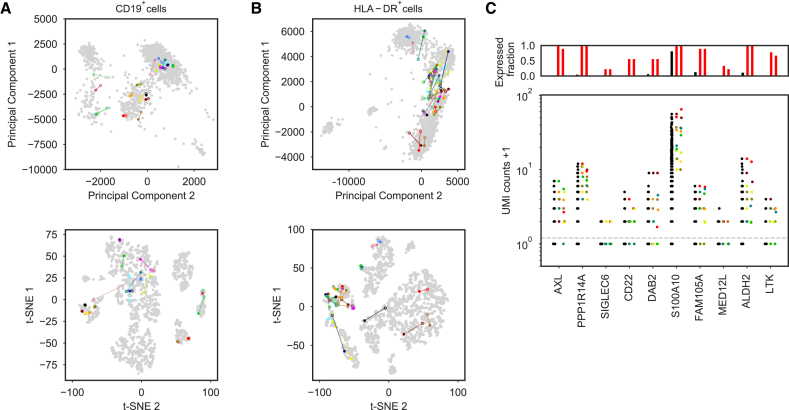
Single-cell expression profile before and after enrichment. Reduced dimensionality representations of 19 cells from CD19^+^ cells (**A**) and 46 cells from HLA-DR^+^ cells (**B**) showing the position of targeted cells based on the expression profiles from the original deep sequenced library (closed circles) and the enriched library (open circles), where each color represents one cell/barcode. The gray data points show all cells within the two original deeply sequenced libraries and make visible the major clusters of cells with related expression profiles. Principal components analysis (PCA) and t-SNE, a nonlinear dimensionality reduction approach, are used to represent the high-dimensional datasets with corresponding color schemes. (**C**) AS dendritic cell signature analysis. Bar plots at top show the fraction of cells with at least one UMI count for the corresponding gene (number of cells above the dashed gray line in bottom panel, with black for all non-target cells, and red for target cells in the original library, middle bar, and red for target cells in the enriched library, right bar). Bottom panel: the expression of the classifier genes for all non-target cells in the original library (black points, left group for each gene), target cells in the original library (colored points, middle group), and enriched target cells (colored points, right). The same color is used for each targeted cell across the different classifier genes to facilitate comparison. The total number of cells in the HLA-DR^+^ cells library was 2397 cells and nine putative AS DC cells were targeted for enrichment.

Next, we sought to quantify differences in gene expression profiles before and after enrichment with principal components analysis (PCA). Post-enrichment expression profiles localized cells to similar locations as found in the original libraries in principal components space when we projected post-enrichment data onto the principal components defined using the original dataset (Figure 2A, B and Supplementary Figure S8). We used Euclidean distance as a metric to quantify how much the position of cells shifted relative to the underlying distribution of cell locations (Supplementary Figure S9). Data clustering by k-means resulted in the same cluster assignments for most cells before and after enrichment (16/19 for CD19^+^, adjusted mutual information score (AMI) = 0.81; and 43/46 for HLA-DR^+^, AMI = 0.75, where AMI = 0 indicates the expected score for random re-clustering, and AMI = 1 indicates identical re-clustering).

### Marker gene expression profiles for AS DCs are reproduced with good fidelity

We then applied this framework to target putative AS DCs by combining enrichment of HLA-DR^+^ cells by FACS with PCR-based multiplexed molecular enrichment from a pooled RNA-seq sequence library to target the extremely rare AS DCs. In the enriched library, only 1 million reads were needed to reliably identify key discriminating genes (11) expressed in the nine putative AS DCs captured in the enriched HLA-DR^+^ library (Figure 2C). Expression of these AS DC-discriminating genes were either not detectable or showed in extremely low counts at the same level of sequencing effort in the original library, which was enriched only by FACS (Supplementary Figure S10). While the biological role of AS DCs remains to be fully elucidated, the discovery study (11) reported several properties relevant to the design of new therapeutic and vaccination modalities, highlighting the need to develop new strategies to enrich and profile rare cell populations like the AS DCs from many different samples to further decipher their unique properties.

## DISCUSSION

Our results demonstrate that individual cells can be enriched at the molecular level from complex pooled single-cell libraries and that the enriched libraries faithfully represent the targeted cells’ original expression profiles. Our PCR approach for targeted enrichment requires a single-cell sequencing library where cell origin is identified by a short barcode sequence, a list of barcode sequences that corresponds to cells of interest, and a set of PCR primers that complement the listed barcodes. Currently, investigators can select cells to target based on initial analysis of a shallow sequence dataset. For many cases, as few as 1000–5000 RNA-seq reads per cell are sufficient to identify cell types of interest (18–21). In other cases, where target cells can only be identified by signatures reliant on detecting the expression of low-abundance transcripts, desirable target cells can be enriched by depleting cells identifiable as other, non-target cell types and low-quality cells (e.g. those with fewer detected UMIs). Approaches that target signature genes specifically would be highly efficient for the positive identification of target cells defined by the expression of low-abundance transcripts, (22–24).

Although the noise sources in aggregate do not have a significant effect on the precision of the expression profiles obtained from the enriched libraries (Figure 1D, Supplementary Figure S4), modifications to the barcode and UMI sequences would enable these noise sources to be further suppressed. In our work, the cell barcode targeting primer had complementarity to the full 16 base pair sequence allowing for the greatest target cell specificity. Lengthening the barcode sequence to add downstream bases that extend beyond the 3′ terminus of the enrichment primer (or alternatively, shortening the enrichment primer) would allow the extension reaction to pick up a portion of the target cell barcode from the library molecule independent of primer hybridization. Extending the length of the UMI sequence, hence its complexity, would increase the average distances between UMI sequences in the final read set and enable more stringent sequence filtering procedures to exclude erroneous reads. Primer modifications, such as 3′ phosphorothioate linkages, could help maintain barcode fidelity and be combined with other design changes. Lastly, while we recommend 25 cycles of PCR in the enrichment PCR, optimization to fewer PCR cycles could potentially improve the quality of enriched sequence libraries when the input library is of high quality and contains a sufficient fraction of on-target content.

Target enrichment is most advantageous when targeting rare populations and the potential enrichment-fold achievable by targeting is large. In this work, we utilized individual oligonucleotide primers to enrich the target cells, which is convenient for targeting small numbers of cells as would be needed for rare population studies. To explore the tradeoff in sequencing effort and the need for primer synthesis, we plotted results from a simple model representing a typical contemporary use case as a function of the abundance of the target cell population (SI and Supplementary Figure S11). Within the assumptions of our model, targeting is favorable for target cell abundances as high as 5%. Emerging advances including those in small custom oligonucleotide primer pool production are likely to accelerate PCR enrichment workflows and make PCR enrichment practical for target populations at abundances >5% by reducing the cost per custom primer (25). In addition, technologies and approaches for pooled single cell library construction are improving rapidly (26) which promise to make sequencing, rather than pooled sample preparation, the overall workflow bottleneck, and bring attention to the need for cell targeting approaches. Our enrichment protocol depends primarily on the presence of cell-specific barcodes and is readily extensible to a wide variety of pooled single-cell applications beyond expression profiling that are read out using DNA sequencing and encode cell of origin using a compact sequence barcode (27–29). Compatible scRNA-seq approaches include 10X Genomics (used here), Drop-seq, and Seq-well. Further development of the protocol described here or alternative approaches would be required for applications that distribute the cell identity information more sparsely across the library molecules, for example those that use dual end barcoding or long barcodes.

Importantly, target cell enrichment may have future biomedical applications. For example, our enrichment method may allow comparison of rare cell types across cellular mixtures from many subjects, such as tracking rare malignant cell states, non-malignant cell states in tumor samples, and circulating tumor cells (CTCs) in blood. In-depth analyses of particular cells of interest may enable access to more precise single-cell expression profiles and enable diagnostic, prognostic, or theranostic tests informed by quantitative (rather than binary) gene expression states that are invisible to current analytics like flow cytometry or imaging. Targeted molecular enrichment of target cells from large pooled single-cell sequence libraries promises to reduce the sequencing effort required to profile rare cells by one to two orders of magnitude while simultaneously enabling selective deep sequencing of high-information-content cells.

## DATA AVAILABILITY

The custom scripts used for secondary analysis can be found at (https://github.com/nranu/SC_enrichment).

Processed scRNA-seq data are available through the Gene Expression Omnibus, (GSE116683).

Raw RNA sequencing data are available through Database of Genotypes and Phenotypes (dbGaP), accession number phs to be assigned.

## Supplementary Material

Supplementary Data
