# Supplementary material for: Targeting individual cells by barcode in pooled sequence libraries
Source: Nucleic Acids Res. 2018 Sep 26;47(1):e4. doi: 10.1093/nar/gky856 (PMC6326790; doi:10.1093/nar/gky856)
Supplement: Supplementary Data [file gky856_supplemental_files.pdf]

Supplemental Information

Target cell enrichment calculation

In Figure 1B, the enriched and control datasets were down sampled to identify the number of total genes identified at various read depths. Reported enrichment fold values were calculated by determining the fold-difference in overall sequencing effort required to identify expression of 50% of the "detectable genes" for the target cell out of the maximum number of "detectable genes" for a particular cell, defined operationally here as the total number of genes detected in the deeply sequenced original library. We also performed a similar analysis for low abundance UMIs (UMIs with 3 reads in the deeply sequenced pre- library) and found that the enrichment values for low abundance UMIs was slightly lower but similar (also about 100-fold) than found in the analysis for all UMIs, indicating good sensitivity of the method for detecting low-abundance UMIs.

UMI-gene pair uniqueness analysis

To determine the frequency of cases in which UMI sequences "collide" on a given gene and how this was affected by the PCR enrichment procedure, we modeled the expected frequency of UMI collisions and compared these results to experimental data. We detect "collisions" as observations of an identical UMI-gene pair associated with more than one cell. The theoretical distribution of such events is modeled as a Poisson distribution with a mean equal to the ratio of the unique number of UMIs in the original library to the total number of UMIs observed. The UMI-gene pairs that were identified in the enriched cells was then compared to all existing UMI-gene pairs in the deeply sequenced original samples. The sequencing effort was normalized on a per cell basis.

Sequencing cost model

Non-enriched

$$\text{Total Cost} = \frac{(\text{Cost}_{\text{read}}) \times (\text{Reads}_{\text{cell}})}{\text{Abundance}}$$

Enriched

$$\text{Total Cost} = \underbrace{\text{Cost}_{\text{primer}}}_{\text{Primer synthesis}} + \underbrace{\frac{(\text{Cost}_{\text{read}}) \times (\text{Reads}_{\text{cell}})}{(\text{Abundance}) \times (\text{Enrich}_{\text{fold}})}}_{\text{Enriched sequencing}} + \underbrace{\frac{(\text{Cost}_{\text{read}}) \times (\text{Reads}_{\text{cell}})}{\text{Abundance}}}_{\text{Shallow sequencing}}$$

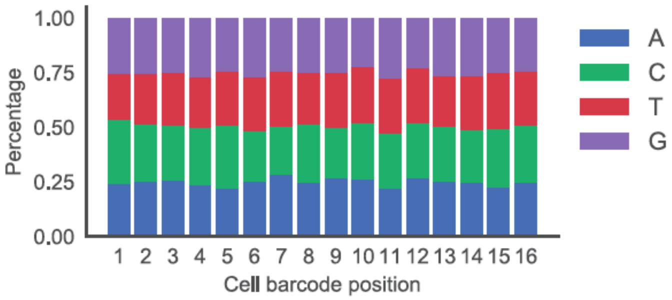

Fig S1. Cell barcode structure. The base composition of each position of the 16 bp 10X cell barcodes in the original HLA-DR library. The GC content per barcode ranges from 31% to 69%.

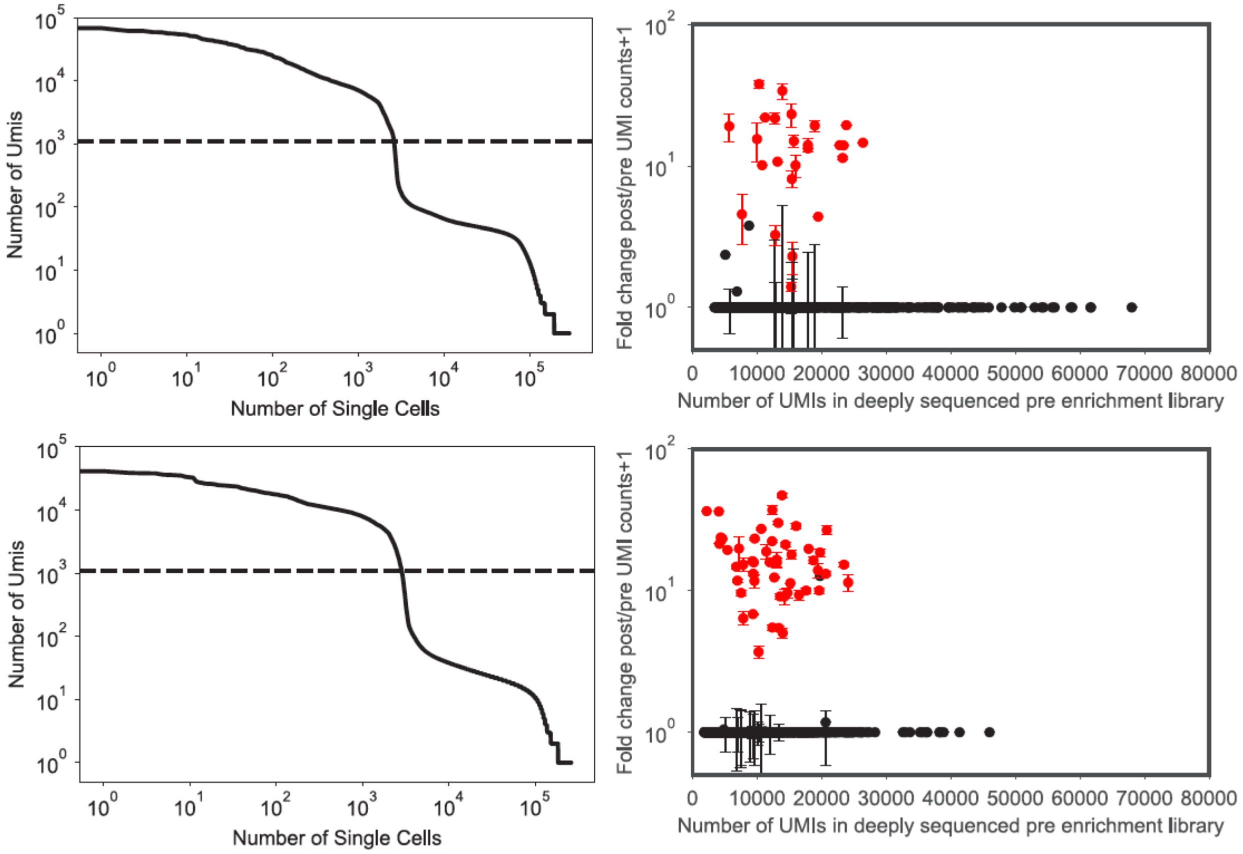

Fig S2. UMI count distribution and fold changes in single cell RNA-seq sequence library of CD19^{+} cells (top) and HLA-DR^{+} cells (bottom). UMI distribution (left). The dashed line (threshold of approximately 1000 UMIs) represents the cutoff used when selecting cells/barcodes for analysis (the cutoff was set using 10X Genomics' CellRanger pipeline). UMI enrichment (right). The number of UMIs detected in the targeted group of cells (red) was increased 10 - 100 fold in the targeted cells/barcodes after multiplexed enrichment. The total sequencing effort for the control and enriched libraries were normalized. The few non-target barcodes seen above are similar in structure on the 5' end to the barcodes that were targeted.

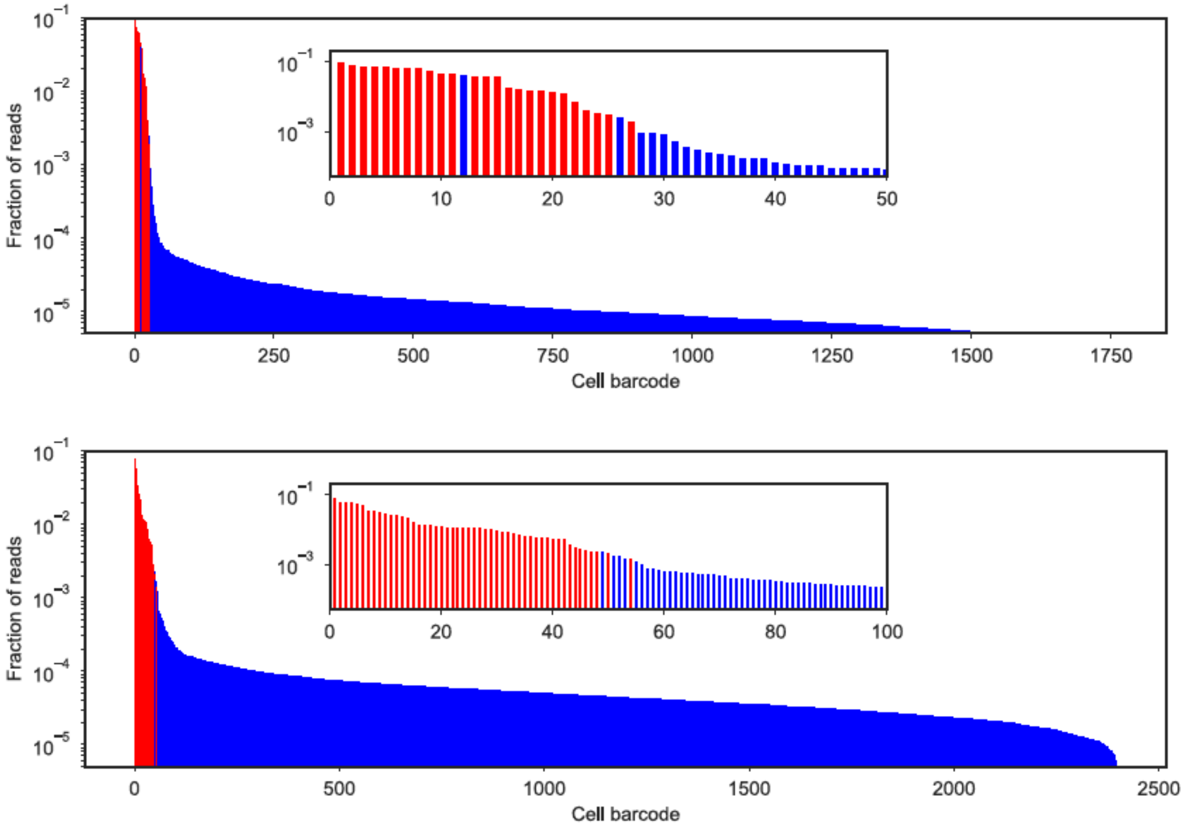

Fig. S3 Analysis of on-target and off-target sequencing reads. Read abundance (data fraction) by cell barcode in the post enrichment dataset for the CD19^{+} (top) and HLA-DR^{+} (bottom) libraries. The data highlighted in red represent on-target amplification whereas the data in blue correspond to other non-target barcodes (originating from the pre enrichment libraries).

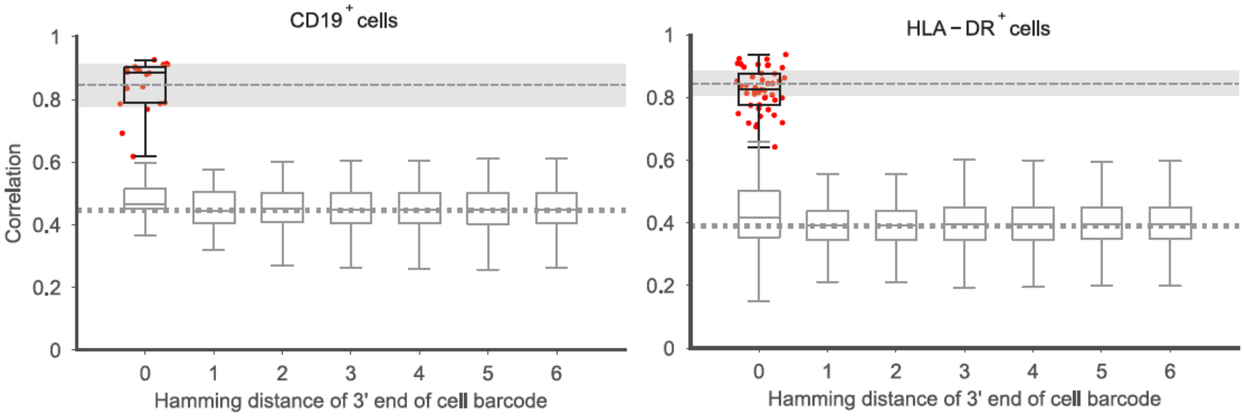

Fig S4. Correlation of single-cell gene expression profiles before and after enrichment. The pairwise correlation of gene expression profiles before and after PCR enrichment all libraries. The upper dashed line and shaded region in each plot represent the mean +/- two standard deviation of bootstrap replicates of the original gene expression profiles against themselves (which represents the best correlation achievable given the read sampling, UMI sampling, and distribution of expression levels across genes in these specific cells). Red points show the correlation for targeted cells (post-enrichment profiles versus pre-enrichment profiles for the same cell). Gray box plots show distribution of correlation coefficients for control (non target) cells existing in the library (post-enrichment profiles of the subject control cell versus pre-enrichment profiles of all cells). The dotted line shows the mean correlation for the cell barcodes that had at least 6 mismatches at the 3' end. Control comparisons are shown as a function of the number of mismatches (Hamming distance) between the six most 3' base pairs of the 16 base pair subject control cell barcode and the six most 3' base pairs of the 16 base pair barcode of compared targeted cells.

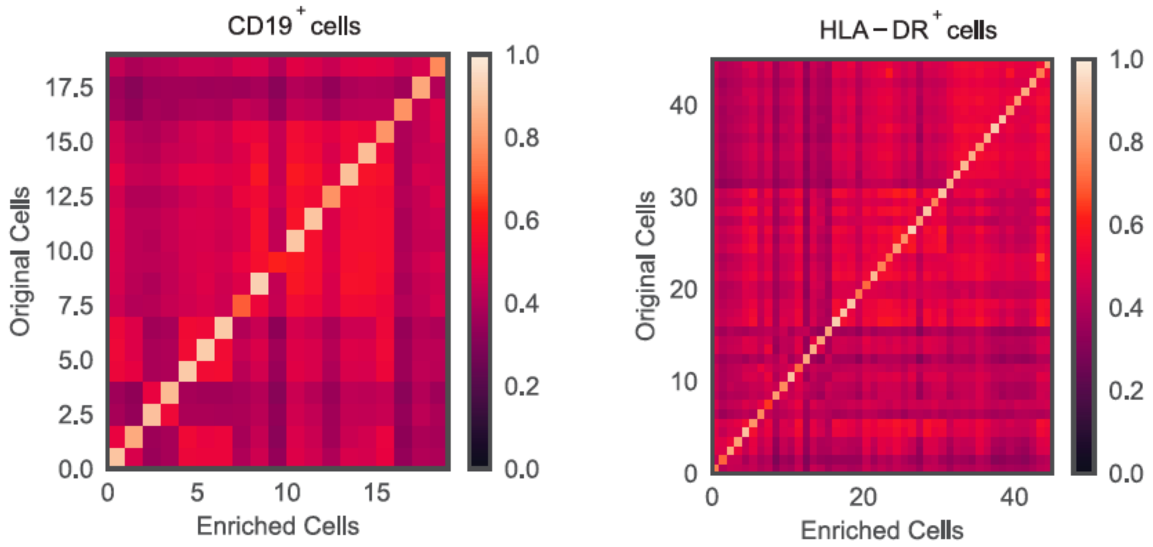

Fig S5. Pairwise correlation of all enriched libraries. The heatmap shows a comparison of the targeted barcode in the control versus the enriched for all libraries. The diagonal represents correlations for targeted cell pre/post PCR enrichment.

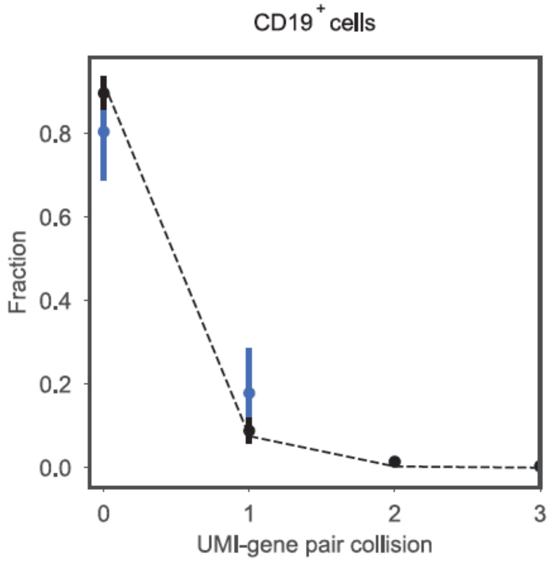

Fig. S6. Distribution of duplicate UMI-gene pairs. The frequency of duplicate UMI-gene pairs across all targeted cells/barcodes. The dotted line represents the expected distribution of UMI-gene duplicates across cells/barcodes. The black points represent the observed fractions of UMI-genes in the pre- enriched sample and the blue points represent the corresponding fractions of all cells in the post- enriched sample. The error bars represent +/- 1 SD across all cells.

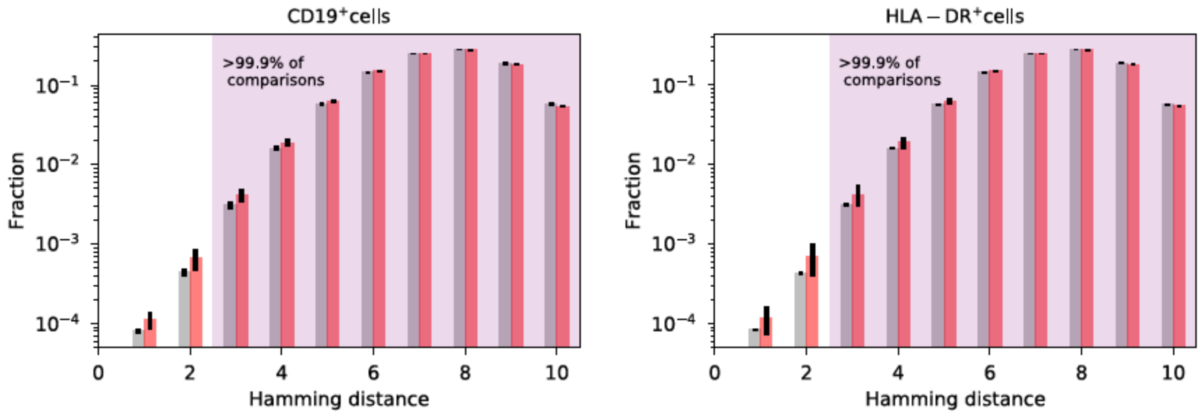

Fig. S7 Analysis of pre and post enrichment inter-UMI distances. The histogram of the pairwise hamming distance between all UMI families identified for each cell barcode. The data show the mean and standard deviation of hamming distances across all cell barcodes for $CD19^{+}$ (left) and HLA-DR^{+} (right) libraries. The distribution of hamming distance is shown for both the pre (gray) and post (red) enrichment datasets.

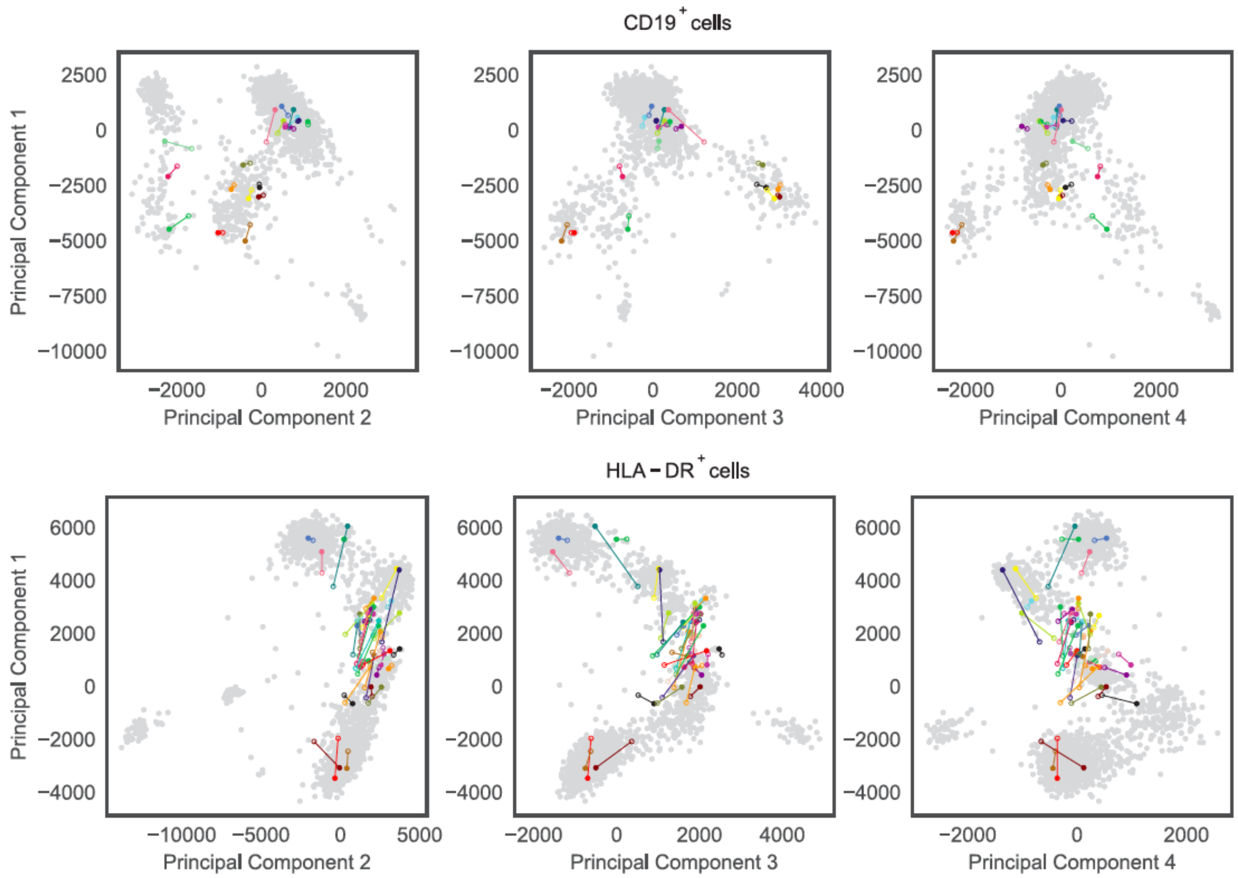

Fig S8. Principal components analysis (PCA) of each multiplex target enrichment set from RNA-Seq sequence libraries of CD19$^+$ cells and HLA-DR$^+$ cells. Each row plots a sample library that was enriched (PC1 versus PC2, PC3, and PC4 in the columns). Closed circles and open circles refer to pre- and post-enrichment positions respectively, with each color representing a single cell.

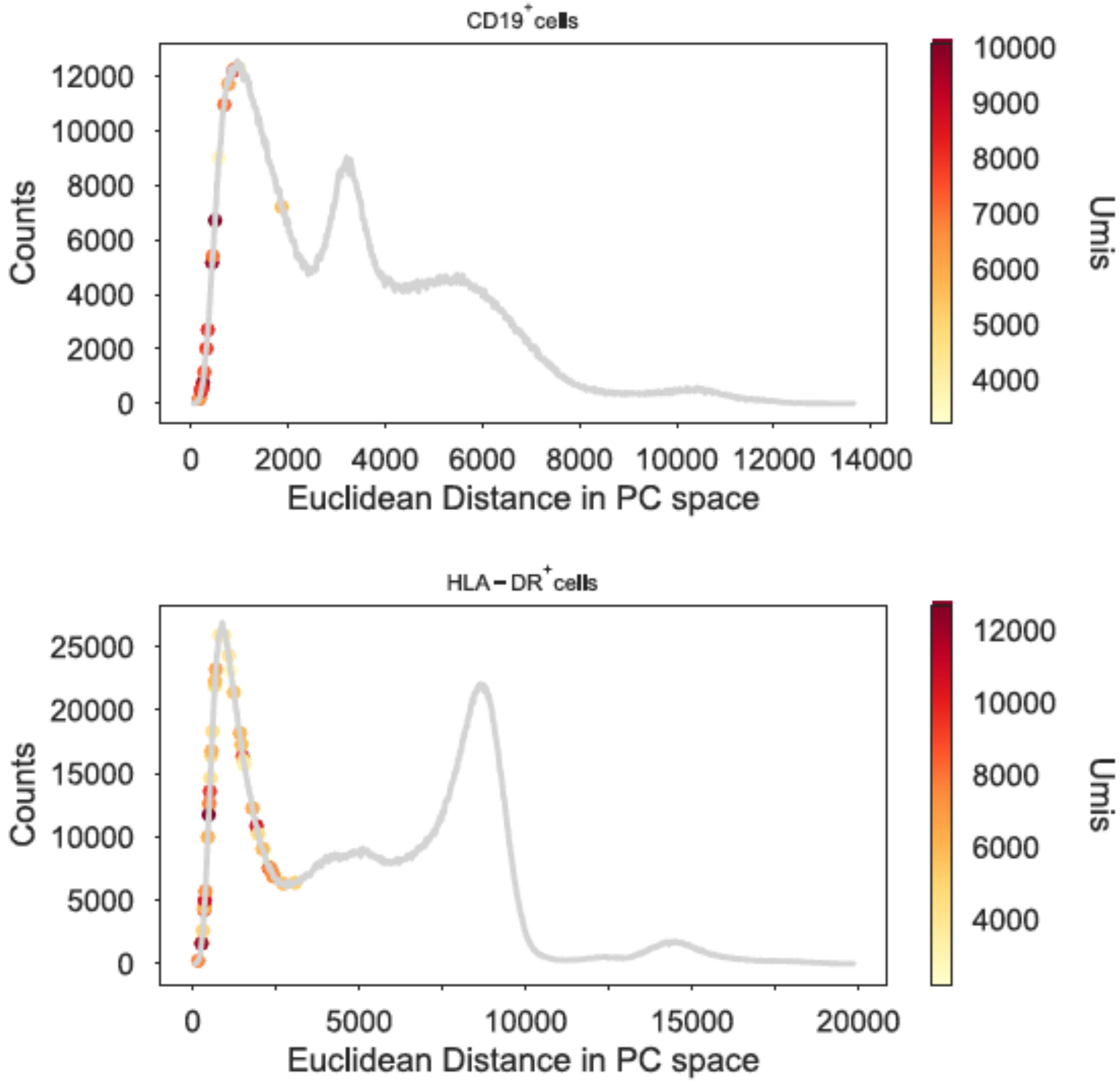

Fig S9. Pairwise Euclidean distance of all targeted barcodes pre/post enrichment in principal components (PC) space. $CD19^{+}$ cells (top) $HLA\text{-}DR^{+}$ cells (bottom). The histogram represents the distance between all single cells in the original, pre- enriched library (deeply sequenced). The colored points on the histogram represent each of the cells/barcodes enriched, which are shaded according to the number of UMI counts detected in the original, pre- enriched library.

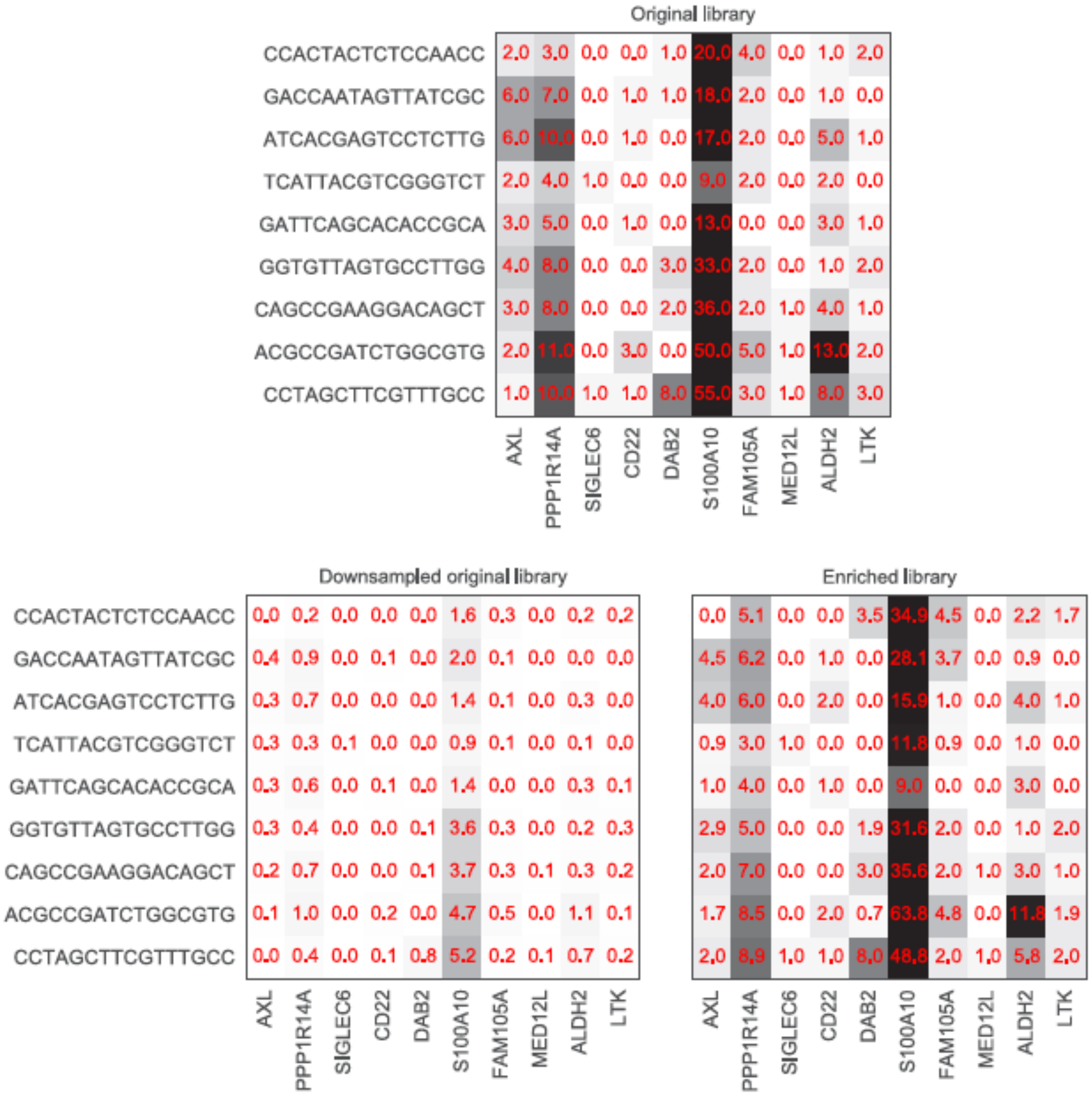

Fig S10. Gene expression profile of the previously described 10 best classifier genes for AXL⁺SIGLEC6⁺ (AS) DCs. The UMI counts used to generate Fig. 2C. A comparison of gene expression profiles for marker genes defining AS DCs between the deeply sequenced control library (top) and the enriched samples (bottom right). Sequencing the original library at equal depth to the enriched libraries shows only low level expression (bottom left).

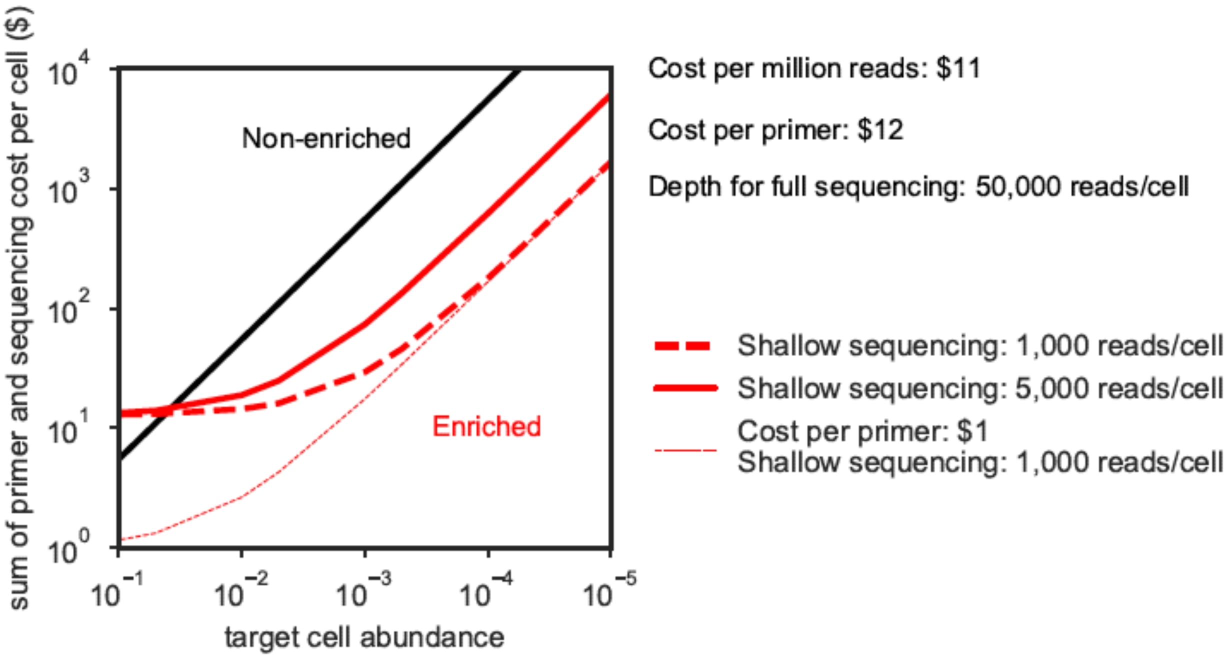

Fig. S11. Analysis of sequencing/primer synthesis trade off at different target cell abundances. The sequencing cost per cell is plotted for enriched libraries (red) and the original library (black) with target cell abundances ranging from 10% to 0.001% and the assumptions as listed. Lower read counts per cell in the initial shallow sequencing step for target identification correspond to 1) whole transcriptome profiling to identify target cell types characterized by profiling high-expression genes (1,000 reads per cell) and 2) gene enrichment to identify target cell barcodes characterized by low-expression genes (1000 reads per cell) [1]. Lower primer synthesis cost corresponds to hypothetical future cases where 1) individual primers are pre-ordered and inventoried by a commercial provider or research organization such that primer costs can be amortized across multiple enrichment runs and 2) small custom primer pools are available as a service.

CD19+ cells

Single cell Barcode	nUMIs in pre	nGenes in pre	nReads in Enrichment	PCR plex	Enrichment Fold	Correlation	Notes
AAATGCCTCATGGTCA	7858	2315	824042	10	110.61	0.90	
ACGATACTCCTATTCA	8144	2324	91965	10	12.08	0.84	
AGACGTTAGGAGTTGC	6384	1868	171784	10	44.07	0.89	
CATATTCAGCCAGTTT	6178	1868	592900	10	143.34	0.88	
CGCTATCGTCCAAGTT	9518	2223	1043853	10	120.26	0.91	
CTCACACGTAGGACAC	7971	2010	501911	10	85.71	0.91	
GATGCTACACTTACGA	10052	370	691070	10	246.92	0.52	Low gene in pre
GGCCGATAGTGTCCCG	9779	2236	853049	10	125.90	0.91	
AGGGAGTAGAAACCGC	5411	143	209971	10	109.18	0.67	Low gene in pre
GATGCTACACTTACGA	10052	370	691070	10	244.77	0.52	Low gene in pre
GCAAACTAGTCAAGGC	7648	246	470000	10	216.89	0.63	Low gene in pre
GTCAAGTCATGTTGAC	6545	209	152900	10	69.03	0.63	Low gene in pre
CTGTGCTAGATGTCGG	6964	261	41053	10	10.88	0.78	Low gene in pre
CTGAAGTCACTCGACG	9971	388	413623	10	134.74	0.74	Low gene in pre
AACTCAGCACCCATGG	7094	1857	1217407	10	217.70	0.69	
AAGACCTTCGGCGGTT	7507	1966	893563	10	223.75	0.93	
AGAGCTTAGATGAGAG	7965	2007	24269	10	1.94	0.62	
CGATGTACAGCGTTCG	10077	2467	920177	10	153.58	0.90	
CAGGTGCGTGTATGGG	8540	2200	488395	10	86.81	0.90	
CTGGTCTTCTCTTATG	6952	1748	53153	10	7.70	0.79	
CTGATAGGTGGCTCCA	9580	2177	198282	8	49.43	0.89	
GTCTCGTCACACCGCA	8218	2093	602383	8	149.45	0.88	
TTGACTTAGAATCTCC	5497	1524	224032	8	57.80	0.79	
TACCTATTCTTTACGT	3552	1337	38167	8	12.97	0.79	
TAAGAGAGTACCTACA	3701	1446	2080	8			Low reads in enriched
GAGCAGAGTTGAACTC	6131	1926	134	8			Low reads in enriched
GTCGGGTCACGCGAAA	3233	1383	194051	8	89.16	0.84	
ATAGACCAGTCCGGTC	5906	1656	959804	8	297.35	0.77	

Supplementary Table 1. Summary table for CD19^{+} cell barcode targets. Barcodes identified in red either had low sequencing reads associated after enrichment or low number in genes identified in the original library and were removed from further analysis.

HLA-DR^{+} cells

Single cell Barcode	nUMIs in pre	nGenes in pre	nReads in Enrichment	PCR plex	Enrichment Fold	Correlation	Notes
GCACTCTGTGCCTGCA	1650	38	127674	11			Low genes and UMI in pre
CGTCAGGAGATGCCTT	1650	96	125667	11			Low genes and UMI in pre
GGCCGATGTCGTCTTC	1534	99	139187	11			Low genes and UMI in pre
TTAGGCAAGCACGCCT	1339	491	142003	11			Low genes and UMI in pre
GTTACAGGTACGACCC	2522	992	223089	11	195.33	0.74	
CAGCTAATCCAAAGTC	2208	995	237749	11	165.57	0.71	
TATCAGGCAGGGATTG	4678	1495	330127	11	157.60	0.84	
ATCACGAGTGACCAAG	4602	1496	324238	11	150.10	0.82	
GGGTCTGGTAGCTCCG	7501	1991	275944	11	80.98	0.90	
GCTCCTACAGACTCGC	9079	1996	659647	11	169.20	0.80	
AGTGGGAAGACACTAA	10854	2463	13800	11			Low reads in enriched
AACACGTTCCACGCAG	3198	956	139292	10	97.28	0.78	
TTCTCAAAGGGCACTA	4543	1169	256944	10	109.12	0.64	
ACGTCAAAGTACGATA	6241	2133	55921	10	18.60	0.81	
GAGTCCGCAATCACAC	5667	1900	298236	10	119.37	0.78	
ACAGCCGAGTGAACGC	9969	2473	275116	10	78.10	0.90	
GGTATTGCAATGTTGC	5104	1306	403230	10	184.78	0.72	
TTCTCCTCACGACGAA	3607	1492	59057	10	38.54	0.84	
TGCCAAATCGCACTCT	4401	1460	147491	10	79.98	0.85	
TCAGATGCAGACAGGT	4208	1347	48426	10	21.05	0.82	
TTCCCAGCACACAGAG	3258	1321	276432	10	59.93	0.83	
CCTAGCTTCGTTTGCC	10532	2681	259572	10	55.61	0.92	
ACGCCGATCTGGCGTG	10288	2472	743352	10	111.44	0.72	
GTGAAGGAGGTGATTA	8163	2171	13816	10			Low reads in enriched
CAGCCGAAGGACAGCT	7648	2284	188554	10	51.81	0.90	
GGTGTTAGTGCCTTGG	7331	2114	151393	10	42.52	0.91	
GATTCAGCACACCGCA	3758	1309	73142	10	14.65	0.74	
TCATTACGTCGGGTCT	3382	1245	261260	10	103.57	0.71	
ATCACGAGTCCTCTTG	7875	2148	173557	10	38.32	0.88	
GACCAATAGTTATCGC	7392	2064	516990	10	121.41	0.76	
CCACTACTCTCCAACC	7343	2162	765042	10	164.26	0.72	
AGCGTATTCATTGCCC	10597	2306	1439195	10	148.75	0.82	
GCTTGAAAGTGAAGAG	10088	2367	334239	10	32.20	0.79	
CGCGGTATCGGCATCG	12141	2706	796027	10	79.35	0.94	
AGAATAGGTCTAGGTT	7664	2081	8679	10			Low reads in enriched
CACAGTATCAATACCG	4593	1302	32994	10	4.24	0.81	
TCAACGATCGCACTCT	9554	2323	613668	10	72.40	0.91	
CCACTACTCTCTAGGA	5895	1560	268203	10	37.30	0.76	
TCTCATAGTGGTCCGT	12695	2815	304674	10	26.21	0.90	
CGATGGCAGTCCATAC	3594	1364	273390	10	65.42	0.85	
GTGAAGGCAATGGAGC	6101	2018	572219	10	85.45	0.86	
GTCTCGTAGCACAGGT	5814	1874	639242	15	81.28	0.88	
GCAGTTAAGGGTCGAT	7257	2169	760696	15	93.80	0.86	
GGGAGATCAAGCGCTC	6405	1898	1422742	15	211.93	0.80	
GGCGTGTAGATCGGGT	1939	911	277	15			Low reads in enriched
GCCAAATTCAGTCCCT	5789	2052	1502496	15	224.65	0.87	
CTACACCGTCAAGCGA	9097	2361	199714	15	20.39	0.92	
AACCGCGTCGAATGGG	5026	1726	90286	15	19.87	0.91	
TACGGATTCAATCTCT	6191	2001	123374	15	11.32	0.80	
TTCTTAGGTTACGTCA	6729	2018	67294	15	5.44	0.81	
TTGTAGGGTTAAAGTG	6066	1910	54834	15	6.75	0.84	
TTCTCCTCACATAACC	5574	1776	208947	15	29.51	0.84	
ACACCAATCTCCAACC	6442	1884	1877930	15	233.53	0.75	
AAAGTAGTCTGCAAGT	8906	2395	4213	15			Low reads in enriched
CCTAAAGAGTGTTAGA	6217	2172	6661	15			Low reads in enriched
CGATGGCCAAGTCTAC	6385	1854	1327900	15	181.55	0.82	

Supplementary Table 2. Summary table for HLA-DR^{+} cell barcode targets. Barcodes identified in red either had low sequencing reads associated after enrichment or low number in genes identified in the original library and were removed from further analysis.

Antigen	Fluorochrome	Clone	Catalogue	Manufacturer
CD19	FITC	HIB19	302206	Biolegend
CD3	PerCP Cy5.5	HIT3a	300327	BD
CD235a	PE/Cy7	HI264	349111	Biolegend
CD4	PE	RPA-T4	300508	Biolegend
CD8	APC	SK1	344722	Biolegend
CD14	APC-Cy™ 7	MφP9	557831	BD
HLA-DR	BV605	L243	307640	Biolegend
CD56	BV711	HCD56	318336	Biolegend
DAPI			422801	Biolegend

Supplementary Table 3: List of antibodies used to enriched for the $LIN^{-}HLA\text{-}DR^{+}$ cell fraction

REFERENCE

[1] LBA. Woodruff, TE. Gorochowski, N Roehner, TS. Mikkelsen, D Densmore, DB. Gordon, R Nicol, and CA. Voigt. Registry in a tube: multiplexed pools of retrievable parts for genetic design space exploration. Nucleic Acids Research, 45(3):1553-1565, 2017.
